# Use of rituximab in idiopathic retroperitoneal fibrosis

**DOI:** 10.1186/s41927-020-00140-9

**Published:** 2020-08-06

**Authors:** Veronika Boyeva, Hatim Alabsi, Michael A. Seidman, Ryan Paterson, Jason Kur, Luke Y. C. Chen, Silvia D. Chang, Mollie Carruthers

**Affiliations:** 1 Department of Medicine, University of British Columbia, 2775 Laurel Street, Vancouver, BC V5Z 1M9 Canada; 2 Department of Radiology, King Abdulaziz University, Al Ehtifalat St, Jeddah, 21589 Saudi Arabia; 3 Department of Pathology, University of British Columbia, 2211 Wesbrook Mall, Vancouver, BC V6T 2B5 Canada; 4 Department of Medicine, Division of Rheumatology, University of British Columbia, 802 - 1200 Burrard Street, Vancouver, BC V6Z 2C7 Canada; 5 Department of Urology, University of British Columbia, 2775 Laurel Street, Vancouver, BC V5Z 1M9 Canada; 6 Department of Medicine, Division of Hematology, University of British Columbia, 2775 Laurel Street, Vancouver, BC V5Z 1M9 Canada; 7 Department of Radiology, University of British Columbia, 2775 Laurel Street, Vancouver, BC V5Z 1M9 Canada; 8 Arthritis Research Canada, 5591 No. 3 Road, Richmond, BC V6X 2C7 Canada

**Keywords:** iRPF, RPF, RTX, IgG4

## Abstract

**Background:**

Retroperitoneal fibrosis (RPF) is characterized by the proliferation of fibrous tissue in the retroperitoneum. The majority of RPF cases are due to idiopathic or IgG4-related disease. Recent studies on IgG4-related disease have shown rituximab to be an effective treatment. The current first-line treatment for idiopathic RPF (iRPF) is glucocorticoid therapy. Relapse rates vary widely in the literature, and DMARDs remain poorly studied. We sought to evaluate the efficacy of rituximab in idiopathic RPF by quantifying changes in iRPF diameter on imaging pre- and post-rituximab therapy and response by lab parameters in 10 iRPF patients.

**Methods:**

We selected 10 patients diagnosed with iRPF and previously treated with rituximab (1000 mg) in two doses approximately 2 weeks apart. Pre- and post-therapy contrast enhanced cross-sectional abdomen and pelvis imaging were compared. In all patients, the thickest portion of the peri-aortic disease was measured in the axial and coronal planes. The presence of acute or long standing back pressure related renal findings were documented. Details of clinical visits including patient demographics and laboratory evaluations were collected pre- and post-therapy. Statistical analysis was performed using a Wilcoxon signed rank test.

**Results:**

The RPF diameter around the aorta before and after therapy decreased from a mean of 15.9 ± 4.9 mm to 10.6 ± 6.1 mm, respectively (*p* < 0.01). The craniocaudal iRPF mean length decreased from 108.6 mm ± 40.4 mm to 90.6 mm ± 45.9 mm (*p* = 0.02).

**Conclusion:**

A comparison of pre and post-rituximab imaging studies revealed a statistically significant decrease in iRPF diameter following treatment with rituximab.

## Background

Retroperitoneal fibrosis (RPF) is a rare disease characterized by the proliferation of fibrous tissue in the retroperitoneum, most commonly surrounding the aorta from the renal vessels to the branching of the iliac arteries [1]. The estimated annual incidence is 1.3/100,000, with a mean age of diagnosis of 64 years and a male to female ratio > 3:1 [2]. RPF has a number of etiologies, which include idiopathic, IgG4-related, infectious, malignant, and drug-induced [1, 3]. However, 75% of cases are of either idiopathic or IgG4-related disease [1, 3, 4], with idiopathic cases accounting for approximately 32% of RPF [4]. The current first-line treatment for idiopathic RPF (iRPF) is glucocorticoid therapy [1, 3]. Treatment failure has been found to be as high as 25% [1, 5], while relapse rates range from 17 to 72% after discontinuation of treatment [5–7]. DMARDs remain poorly studied [1].

Rituximab has previously been demonstrated as an effective treatment for IgG4-related disease [8–10]. A prospective open label clinical trial demonstrated 97% disease response rate at 6 months post-rituximab therapy in 30 IgG4-related disease patients, providing strong evidence that B cell depletion is an effective treatment for IgG4-related disease (IgG4-RD) [8]. Although histopathologically distinct, it is unknown how much pathophysiological overlap there is between idiopathic retroperitoneal fibrosis and IgG4-RD, or even whether many cases of iRPF are in fact IgG4-related but not meeting histopathologic diagnostic criteria. The effectiveness of rituximab in IgG4-related disease was the basis for trialing it in a similar condition.

We assessed the effectiveness of rituximab by quantifying changes in iRPF diameter on imaging pre- and post-rituximab therapy in 10 iRPF patients as well as response by lab parameters.

## Methods

This study was approved by the Clinical Research Ethics Board at the University of British Columbia. Patient charts were reviewed from the period of January 2015 to October 2018 from a single user electronic medical record (EMR). Patients were identified by radiographic findings consistent with retroperitoneal fibrosis and a biopsy consistent with idiopathic RPF. All patients previously received rituximab treatment, and patients that were previously treated with prednisone or disease modifying anti-rheumatic drugs (DMARDs) were included. Patients with clinical, serologic or pathologic evidence of IgG4-related disease were excluded. All patients were actively flaring at the time of treatment. Ten patients were selected based on these criteria, with 9 retrospective patients and one prospective patient who was undergoing rituximab treatment at the time of the study.

Out of the 10 selected patients, all except one patient who declined biopsy had histopathologic proof of their idiopathic RPF diagnosis. Tissue samples were obtained through laparoscopic resection of pathological tissue surrounding the ureter in all 9 biopsies. The biopsies were reviewed by one pathologist who specializes in reviewing RPF cases (MS). Histological confirmation of disease was defined as cases having dense fibrosis with variable amounts of inflammation, predominantly mononuclear (lymphohistiocytic) and/or occasional lymphoid aggregates. These cases lacked evidence of another etiology, i.e. no granulomas, no cytologic atypia or other evidence of malignancy, no overt necrosis, no significant neutrophilic or eosinophilic inflammation, no vasculitis, no significant plasma cell component, no significant storiform fibrosis, no endarteritis or obliterative phlebitis, and no increase in IgG4 positive plasma cells (as defined by IgG4 related disease consensus criteria) [11].

All 10 patients were previously treated with 2 doses of rituximab (1000 mg) approximately 2 weeks apart as part of their routine care. Pre- and post-therapy contrast enhanced cross-sectional abdomen and pelvis imaging, of which 19 were CTs and 1 was an MRI, were compared by radiology (HA, SC). In all patients, the thickest portion of the peri-aortic disease was measured in the axial and coronal planes. The presence of acute and/or long standing unilateral or bilateral back pressure related renal findings were also documented (e.g. hydronephrosis, presence of stents, renal atrophy, and involvement of ureter, renal vessels, or common iliac vessels). Details of clinical visits including patient demographics, symptoms, past treatments, disease duration, biopsies, concurrent treatments were collected pre- and post-therapy (Table 1). Pre-treatment laboratory values for IgG4 levels, albumin, creatinine, CRP, WBC, and hematocrit were also collected. Statistical analysis was performed using the Wilcoxon signed rank test. A probability of *p* < 0.05 was considered statistically significant. The relationship between disease duration and response to treatment was assessed using Spearman’s rank correlation.

**Table 1:** Demographic and clinical characteristics of study patients

Characteristic	No. (%)
No. of patients	10
Age of onset, years^a^	58.5 ± 11.6
Gender	
Male	8 (80)
Female	2 (20)
Presenting Symptoms	
Flank Pain	4 (40)
Abdominal Pain	2 (20)
Nausea	2 (20)
Incidental Finding	2 (20)
Back Pain	1 (10)
Constipation	1 (10)
Weight Loss	1 (10)
Polyuria	1 (10)
Baseline Lab Parameters	
IgG4 (g/L)	0.1–4.6
CRP (mg/L)	1.7–44.1
WBC (10^9^/L)	8.9 ± 2.5
Hematocrit (L/L)	0.42 ± 0.36
Albumin (g/L)	40.0 ± 2.7
Creatinine (umol/L)	105.3 ± 19.7
Biopsy Proven Disease	
Idiopathic RPF	9 (90)
Biopsy Declined	1 (10)
Past Treatment History	
Prednisone	4 (40)
Azathioprine	3 (30)
Disease Duration Pre-Rituximab, months^a^	17 ± 13
Concurrent Treatment with Rituximab	
Prednisone	3 (30)
None	7 (70)

^a^Mean ± SD

## Results

The average age of disease onset was 58.5 ± 11.6 years (Table 1). Eight of 10 patients were male and only 2 were female. The most common symptom at presentation was flank pain, followed by abdominal pain, nausea; some patients’ retroperitoneal fibrosis was identified incidentally. Prior to rituximab, 4 patients were treated with at least one course of prednisone. Three of the 4 patients who were previously treated with prednisone continued the treatment concurrently with rituximab. Three patients were previously on azathioprine 100 mg, with all 3 developing significant side effects resulting in discontinuation of this treatment within 5–6 weeks of commencement.

On those patients where biopsies were available, there was a varying degree of fibrous thickening and disruption of the connective tissue. Although, there was a varying degree of inflammation and some cases with occasional lymphoid aggregates. As fully described in the Methods, there were no features of malignancy or infection, none of the cases met consensus criteria for IgG4-related disease, and no other apparent etiology (e.g. sarcoidosis, vasculitis, etc.) was identified.

**Fig. 1:**
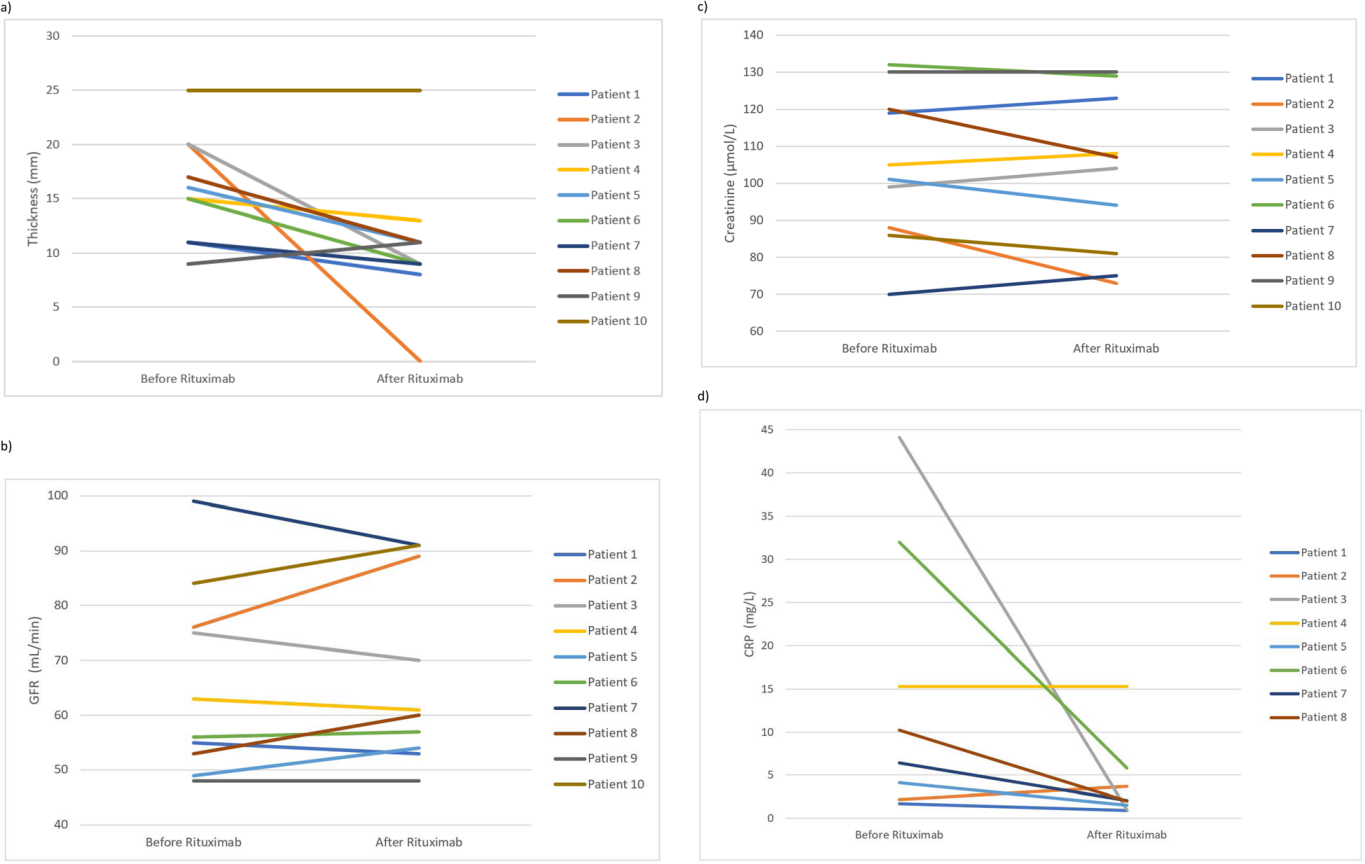
Changes in measured parameters pre- and post-rituximab therapy **a**) Maximal Thickness Around the Aorta: Before and After Rituximab **b**) GFR Before Rituximab and After Rituximab **c**) Creatinine Before Rituximab and After Rituximab **d**) C Reactive Protein Before Rituximab and After Rituximab. Patients 2, 3, and 7 were on concurrent prednisone

The GFR increased from 65.3 ± 16.0 to 67.4 ± 16.8, the creatinine decreased from 105.3 ± 19.8 to 102.4 ± 21.3, and the CRP decreased from 14.5 (1.7–44.1) to 4.0 (0.9–15.3) when comparing mean values before to after therapy, respectively, although none of these results met statistical significance in this cohort (Fig. 1).

**Fig. 2:**
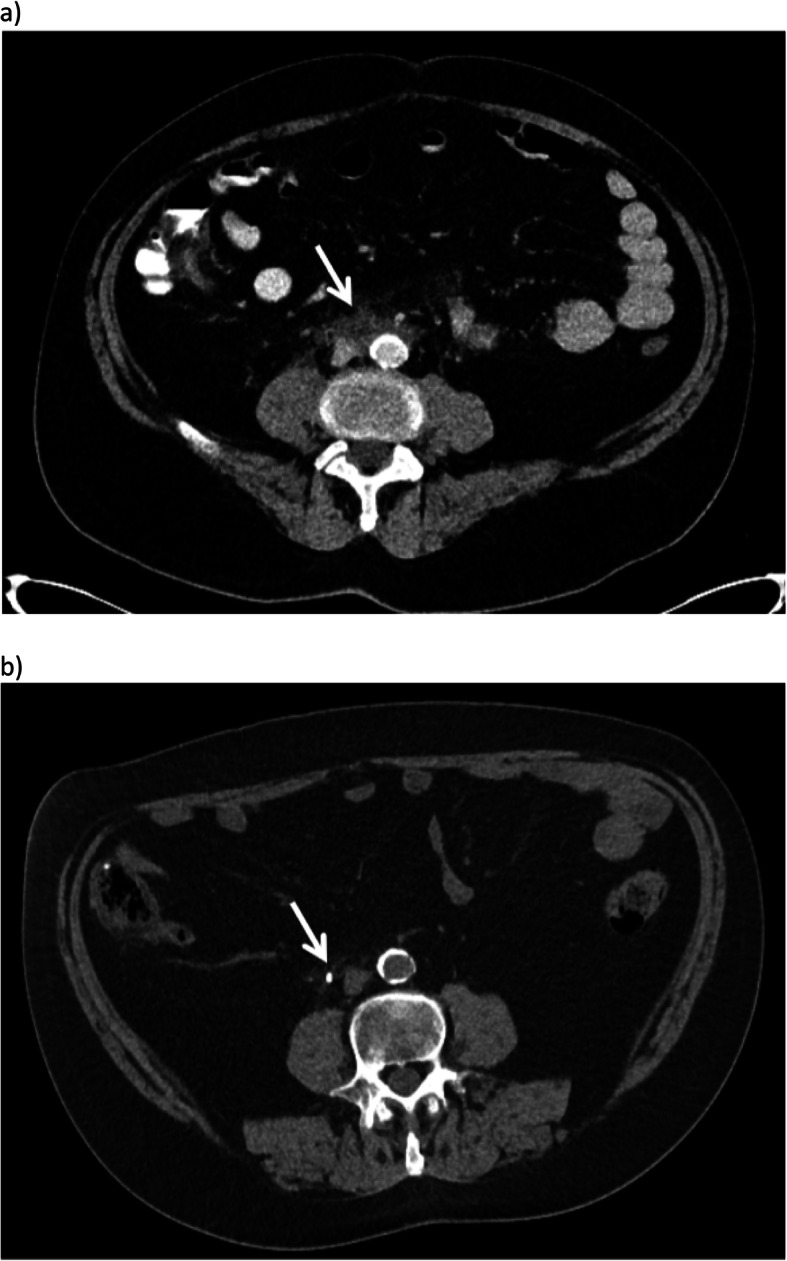
CT scan of Patient 2 pre-treatment (**a**) demonstrating a soft tissue mass (arrow) in keeping with RPF partially encasing the aorta, IVC and the right ureter. CT scan performed subsequently in the same patient post treatment (**b**) demonstrates that the soft tissue representing RPF has almost completely resolved. There is only a small amount of residual tissue (arrow) surrounding the right ureter, which has a stent in place

A comparison of pre and post-rituximab imaging studies were available in 10 patients and revealed statistically significant improvements in iRPF diameter following treatment with rituximab on imaging in the axial and coronal planes (Table 2). The RPF diameter around the aorta before and after therapy decreased from a mean of 16.1 ± 4.6 mm to 10.4 ± 6.2 mm, respectively (*p* < 0.01), as shown in Fig. 1. The craniocaudal iRPF mean length decreased from 108.6 mm ± 40.4 mm to 90.6 mm ± 45.9 mm (*p* = 0.02). Figure 2 demonstrates marked improvement on comparison of pre and post-imaging of one patient. All pre and post-treatment imaging were CTs, with the exception of one post-treatment MRI. Pre-treatment imaging was done, on average, 6.5 months (mean) prior to rituximab therapy, and post-treatment imaging was completed 5 months (mean) after treatment.

**Table 2:** Changes in parameters assessed on imaging, pre- and post- rituximab

Parameter:	Pre-Rituximab Treatment No. (%)	Post-Rituximab Treatment No. (%)	*P* Value
Thickness of RPF Mass^a^ (mm)	16.1 ± 4.6	10.4 ± 6.2	0.01
Craniocaudal RPF Length^a^ (mm)	108.6 ± 40.4	90.6 ± 45.9	0.02
Presence of Hydronephrosis	7/10 (70)	4/10 (40)	0.37
Unilateral	5/10	3/10	
Bilateral	2/10	1/10	
Presence of Renal Atrophy (unilateral)	5/10 (50)	6/10 (60)	1
Presence of Renal Stents	1/10 (10)	4/10 (40)	0.3
Unilateral	1/10	3/10	
Bilateral	0/10	1/10	
Ureter Involvement	10/10 (100)	10/10 (100)	1
Unilateral	3/10	5/10	
Bilateral	6/10	5/10	
Renal Vessel Involvement	3/10 (30)	3/10 (30)	1
Unilateral	1/10	2/10	
Bilateral	2/10	1/10	
Common Iliac Vessel Involvement	10/10 (100)	10/10 (100)	1
Unilateral	1/10	2/10	
Bilateral	9/10	8/10	
Imaging Type			
CT	10/10	9/10	
MRI	0/10	1/10	

^a^ Mean ± SD

Pre-rituximab, 7 of 10 patients had renal hydronephrosis on imaging, while 4 out of 10 patients had hydronephrosis post-treatment (*p* = 0.37). Five of 10 patients had unilateral renal atrophy on initial imaging and 6 of 10 patients had atrophy on post-rituximab imaging. The number of patients with renal stents increased from 1 out of 10 to 4 out of 10 post-therapy (*p* = 0.3). All 10 patients had ureter involvement before and after rituximab, with unilateral involvement increasing from 3 patients to 5 patients, and bilateral ureter involvement decreasing from 6 patients to 5 patients. Renal vessel involvement remained similar pre and post-rituximab, with 3 out of 10 patients showing involvement on imaging. Similarly, common iliac vessel involvement was present in all 10 patients both pre and post-treatment, although 1 patient who initially had bilateral iliac vessel involvement had only 1 sided involvement post-rituximab. Infrarenal IVC involvement was present in all 10 patients pre-treatment, and 9 patients post-treatment. There was a moderate correlation between greater percent reduction of RPF in the axial plane and shorter disease duration prior to treatment with rituximab (Spearman’s coefficient = 0.58), and a weak correlation between greater percent reduction of RPF in the craniocaudal plane and shorter disease duration prior to rituximab (Spearman’s coefficient = 0.25).

Symptomatically, 5 of the 8 symptomatic patients reported some improvement in symptoms. Three of the 4 patients who initially presented with flank pain reported at least some improvement in symptoms.

Eight patients tolerated the rituximab infusion without any side effects. One patient developed hives despite administration of diphenhydramine and was unable to complete the first infusion. He was later able to complete both infusions utilizing a lower infusion rate and different pre-medicines. One other patient developed throat irritation and rhinorrhea at their second round of rituximab infusions but was able to finish the infusion following administration of diphenhydramine.

## Discussion

In summary, idiopathic retroperitoneal fibrosis, as distinguished from histopathologically documented IgG4-related retroperitoneal fibrosis, appears to have a similar response to rituximab treatment. There was a non-statistically significant improvement in the CRP, GFR, and creatinine values post-therapy, potentially attributable to such a small sample size. When comparing iRPF diameter on imaging pre and post-rituximab, there was a statistically significant reduction in both axial and coronal planes. It is unknown how the efficacy of rituximab compares to prednisone alone, DMARDs or placebo in this observational case series.

A more recent study of rituximab therapy in RPF looked at 26 patients, 7 of whom were diagnosed with iRPF [12]. The remaining 19 patients had IgG4-RD. Treatment response was defined as either improvement in RPF symptoms, shrinkage of RPF on imaging, or resolution of laboratory markers (IgG4, CRP or ESR). On post-treatment imaging, 21 of the 25 patients (84%) had treatment response, as defined by improvement in RPF size in at least 2 imaging planes. This study, however, chose not to delineate improvement in the iRPF patient population from those with IgG4-RD. Another recent study by Urban *et. al.* [13] looked at 20 iRPF patients with relapsing/refractory disease or with contraindications to glucocorticoids, of which 8 patients were biopsy proven. At 6 months following treatment with rituximab, a significant reduction in periaortic and peri-iliac thickness, and CRP was found. These findings are consistent with those of our study, although our CRP reduction did not reach statistical significance, likely due to a small sample size. This study also found a statistically significant reduction in ureteral involvement. In our study, pre and post-rituximab imaging was used to determine the number of patients with ureter involvement, vessel involvement, and renal stents. The pre-treatment imaging was often done months prior to start of treatment, and therefore may not have accurately reflected the true ureter and vessel involvement prior to treatment. On chart review, it was found that the patients had less ureteral stents following rituximab (3 unilateral, 1 bilateral) than prior to treatment (2 unilateral, 2 bilateral), which suggests that the imaging data likely overestimated the progression of ureter involvement despite treatment with rituximab.

We noted several interesting trends in our study. Firstly, there was one patient who was previously treated with steroids and still benefitted from rituximab therapy, with further improvement seen on imaging post-treatment. It was also noted that 3 patients were on concurrent prednisone at time of RTX treatment. Two of these patients had the best radiological response post-RTX, with 1 achieving complete resolution on imaging, while the other improved by over 50% when reviewing maximal aortic thickness on post-treatment imaging. The third patient on concurrent steroids, who had been successfully treated with steroids in the past, had a less impressive improvement in RPF thickness, of approximately 18%.

Based on this study, there appears to be a role for rituximab in treating idiopathic RPF. While there were statistically significant changes in disease on imaging post-treatment, the clinical improvement of patients was more difficult to assess. Although patients seemed to have some improvement based on clinical records, it is our suspicion that RPF may cause lumbosacral plexus nerve damage given the proximity of those nerves that may result in chronic pain. More studies are needed to assess patients’ long-term outcomes.

This study focused on rituximab as a treatment for idiopathic RPF and did not explore the efficacy of disease modifying antirheumatic drugs (DMARDs) in treating this condition. There is likely a role for DMARDs in idiopathic RPF, but this is yet to be established through further research. One strength of rituximab that was noted in this study was its favorable side effect profile. Only two patients had an allergic reaction during their infusion, but this was quickly mitigated with diphenhydramine and both patients were able to complete treatment. The patient who developed a more severe reaction initially took prednisone 50 mg orally in the evening prior to the first infusion. During the infusion, the patient received diphenhydramine 50 mg IV over 2–3 minutes followed by hydrocortisone 100 mg IV to mitigate the allergic reaction. Prior to the second infusion, this patient took the prednisone 50 mg orally in the morning prior to infusion and was then able to tolerate the infusion well.

There were several limitations to this study. As RPF is a fairly rare condition, the study was only able to include 10 patients who met iRPF criteria. Patients were ruled out to have IgG4-related disease using the histopathological classification by Deshpande *et. al.* [11]. Since the writing of this manuscript, new consensus guidelines have been published [14]. As a retrospective study, there was no opportunity to have a control group of iRPF patients. Future research comparing iRPF patients on prednisone to patients treated with rituximab would provide valuable information on the efficacy of rituximab in comparison to the current standard first-line treatment. There was variation in disease duration prior to treatment as well as previous therapies that were trialed prior to rituximab. In addition, this study included patients who were previously treated with prednisone, as well as patients on concurrent prednisone. Therefore, the effects of prednisone versus rituximab are not fully clear. Again, future studies that include comparison of prednisone to rituximab are needed to further understand the roles of prednisone and rituximab in treating iRPF.

Some questions about rituximab in iRPF still remain. At this time, it is unclear how long to continue treatment with rituximab for iRPF patients who have seen benefit from treatment and when the medication can be stopped. The most feared complication of untreated retroperitoneal fibrosis is end stage renal disease [11]. Re-treatment should be considered with the goal of preventing persistent hydronephrosis and reflux nephropathy. Although stenting and ureterolysis are utilized to treat hydronephrosis, the added role of medical therapy is not known at this time.

There are several strengths to this study. This study specifically selected for idiopathic RPF patients, with most having biopsies showing no evidence of any other etiology. As far as we are aware, this is the largest study to date of rituximab in an iRPF population. The primary outcome, improvement of disease by imaging as an objective measure of disease progression, showed a significant response to rituximab.

## Conclusion

Given the older patient population in iRPF, rituximab may be a steroid-sparing alternative in this glucocorticoid sensitive patient population. Rituximab may also have added treatment benefit for patients who have already been treated with steroids. However, many questions remain, and more studies are needed to establish this medication’s role in treating idiopathic RPF.

## Data Availability

The data used to support the findings of this study are available from the corresponding author upon request.

## Abbreviations

RPF: Retroperitoneal fibrosis
iRPF: idiopathic retroperitoneal fibrosis
RTX: Rituximab
IVC: Inferior vena cava
DMARDs: Disease modifying antirheumatic drugs
EMR: Electronic medical record

## References

[1] Rossi GM, Rocco R, Accorsi Buttini E, Marvisi C, Vaglio A (2017). Idiopathic retroperitoneal fibrosis and its overlap with IgG4-related disease. Intern Emerg Med.

[2] van Bommel EFH, Jansen I, Hendriksz TR, Aarnoudse ALHJ (2009). Idiopathic retroperitoneal fibrosis: prospective evaluation of incidence and clinicoradiologic presentation. Medicine (Baltimore).

[3] Lian L, Wang C, Tian J (2016). IgG4-related retroperitoneal fibrosis: a newly characterized disease. Int J Rheumatic Dis.

[4] Khosroshahi A, Carruthers MN, Stone JH, Shinagare S, Sainani N, Hasserjian R, Deshpande V (2013). Rethinking Ormond’s disease: idiopathic retroperitoneal fibrosis in the era of IgG4-related disease. Medicine..

[5] van Bommel EF, Siemes C, Hak LE, van der Veer SJ, Hendriksz TR (2007). Long-term renal and patient outcome in idiopathic retroperitoneal fibrosis treated with prednisone. Am J Kidney Dis.

[6] Vaglio A, Palmisano A, Alberici F, Maggiore U, Ferretti S, Cobelli R, Ferrozzi F, Corradi D, Salvarani C, Buzio C (2011). Prednisone versus tamoxifen in patients with idiopathic retroperitoneal fibrosis: an open-label randomised controlled trial. Lancet..

[7] Runowska M, Majewski D, Puszczewicz M (2016). Retroperitoneal fibrosis – the state-of-the-art. Reumatologia..

[8] Carruthers MN, Topazian MD, Khosroshahi A, Witzig TE, Wallace ZS, Hart PA (2015). Rituximab for IgG4-related disease: a prospective, open-label trial. Ann Rheum Dis.

[9] Ebbo M, Grados A, Samson M, Groh M, Loundou A, Rigolet A (2017). Long-term efficacy and safety of rituximab in IgG4-related disease: Data from a French nationwide study of thirty-three patients. PLoS One.

[10] Khosroshahi A, Carruthers MN, Deshpande V, Unizony S, Bloch DB, Stone JH (2012). Rituximab for the treatment of IgG4-related disease: lessons from 10 consecutive patients. Medicine (Baltimore).

[11] Deshpande V, Zen Y, Chan JKC, Yi EE, Sato Y, Yoshino T (2012). Consensus statement on the pathology of IgG4-related disease. Mod Pathol.

[12] Wallwork R, Wallace Z, Perugino C, Sharma A, Stone JH (2018). Rituximab for idiopathic and IgG4-related retroperitoneal fibrosis. Medicine..

[13] Urban ML, Maritati F, Palmisano A (2020). Rituximab for chronic periaortitis without evidence of IgG4-related disease: a long-term follow-up study of 20 patients. Ann Rheum Dis.

[14] Wallace ZS, Naden RP, Chari S, Choi H, Della-Torre E, Dicaire JF (2020). The 2019 American College of Rheumatology/European league against rheumatism classification criteria for IgG4-related disease. Arthritis Rheumatol.

